# Transplantation during the COVID-19 pandemic: nothing noble is accomplished without danger

**DOI:** 10.1186/s12876-020-01401-0

**Published:** 2020-08-06

**Authors:** Gabriele Spoletini, Giuseppe Bianco, Dario Graceffa, Quirino Lai

**Affiliations:** 1 General Surgery and Liver Transplantation, Fondazione Policlinico Universitario A. Gemelli IRCCS, Largo Agostino Gemelli 8, 00168 Rome, Italy; 2 Centre for the Study and Treatment of Psoriasis, Department of Clinical Dermatology, San Gallicano Dermatological Institute, IRCCS, Rome, Italy; 3 Hepatobiliary and Organ Transplantation Unit, Sapienza University of Rome, Umberto I Polyclinic of Rome, Rome, Italy

**Keywords:** Coronavirus, Transplantation, Organ donation, SARS-CoV-2, COVID-19, Virus tests

## Abstract

The global health crisis due to the fast spread of coronavirus disease (COVID-19) has caused major disruption in all aspects of healthcare. Transplantation is one of the most affected sectors, as it relies on a variety of services that have been drastically occupied to treat patients affected by COVID-19. With this report from two transplant centers in Italy, we aim to reflect on resource organization, organ allocation, virus testing and transplant service provision during the course of the pandemic and to provide actionable information highlighting advantages and drawbacks.

To what extent can we preserve the noble purpose of transplantation in times of increased danger? Strategies to minimize risk exposure to the transplant population and health- workers include systematic virus screening, protection devices, social distancing and reduction of patients visits to the transplant center. While resources for the transplant activity are inevitably reduced, new dilemmas arise to the transplant community: further optimization of time constraints during organ retrievals and implantation, less organs and blood products donated, limited space in the intensive care unit and the duty to maintain safety and outcomes.

## Background

Since December 2019, the fast spread of the novel Severe Acute Respiratory Syndrome CoronaVirus-2 (SARS-CoV-2) causing a severe acute respiratory disease (COVID-19), has determined a healthcare crisis in a growing number of countries. To date, USA, Spain and Italy have reported the highest number of patients affected, and COVID-19 has been categorized as a global pandemic [1]. Disruptions in almost all aspects of health care provision have been observed, and health systems are trying to continue offering essential services while suspending those that can be postponed.

Transplant services can be categorized depending on their lifesaving nature. Heart, lung and liver transplants are urgent lifesaving operations in a proportion of wait-listed patients. In particular, those with chronic end-stage organ disease who develop deterioration of their baseline condition, and those who suffer from sudden end-stage failure of a given organ.

While it seems obvious that lifesaving transplant activity should not be stopped, it is not clear whether non-lifesaving transplants should be delayed past the most critical phase of the emergency. In fact, prolonging the time spent on the waiting list can translate into waiting list drop-out due to disease progression or overcoming contra-indications.

On May 3, 2020, Italy is the third most affected country worldwide and has registered the second highest number of COVID-19-related deaths so far. The Italian National Authority for Transplantation released guidance on donor and recipient testing for SARS-CoV-2 [2, 3]. Testing via naso-pharyngeal swab (NPS) or bronchoalveolar lavage and, if positive, measurement of viral load on blood sample are recommended in all donors from high incidence regions. SARS-CoV-2 positive potential deceased donors are to be discarded and living donors postponed. NPS is compulsory before transplantation for all potential recipients who are symptomatic or with a history of contact with a COVID-19 positive patient, and discretional for asymptomatic recipients in whom history of contact with COVID-19 positive patient can be reasonably ruled out.

Implications of the spread of COVID-19 for the transplant community are innumerable, and the unprecedented nature of the pandemic has left physicians without guidance in many of their management choices. Balancing resource constraints, patient safety and life-saving organs demand is difficult during COVID-19 pandemic. With the present report we aim to reflect on the open challenges for the transplant community. A summary of actions to be undertaken is summarized, reflecting on advantages and dangers related to each (Table 1).

**Table 1:** Summary of issues and actions to be undertaken to mitigate the risks for the transplant population and staff related to COVID-19

Issues and actions	Advantage	Disadvantage
**Screening and risk exposure for transplant staff**		
Extensive screening of transplant staff	Healthcare workers safety Breaking the vicious circle of in-hospital virus transmission	Increased costs More staff quarantined
Travels reduction – regional organs shipping systems	Reduction of contagion to other hospitals from travelling retrieval surgeons	Need to develop a graft exchange system if not in place yet
**Timing and logistics of transplantation**		
Screening of waitlisted patients	Thorough information regarding patients awaiting transplants	Costs Logistics of testing for patients currently out-of-hospital
Recipients testing at the time of transplant offer	Lower costs compared to previous action	Delays before transplant start Possible cancellation of recipient’s transplant
Back-up recipient in hospital	Prompt replacement if first candidate tests positive	More complex logistics Anxiety and potential frustration for most back-up patients Increased logistics costs.
Use of machine perfusions to fast-track organ retrieval from unstable donors (applicable only to donors with low-risk COVID-19 history)	Extended preservation time Higher organs yield	Increased costs Aborted procedures if COVID-19 tests return positive
Teleclinics for follow-up of transplant recipients	Avoiding access to hospital out-patient clinics - decreased exposure to infection	Increased risk of missing potentially relevant yet subclinical health problems
**Transplant benefit**		
Revisiting local policies of access to transplantation based on hospital resources availability • Privileging “utility” (recipients with expected better outcomes) • Privileging “urgency” (recipients with the highest need)	Realistic approach to resource allocation between COVID and non-COVID diseases • Less resource consumption (faster ICU turnaround, less blood transfusions, etc.) • Treating the sickest patients only and utilize resources for those in desperate need of transplantation	Further stretching healthcare resources with risk of system collapse • Missing the sickest patients; increased mortality without treatment • Uncertainty regarding mortality effect at the “bottom” of the transplant waiting list

### Screening and risk exposure for transplant staff

Since the beginning of the pandemic, health-workers screening has been advocated as an essential tool for: 1) protecting patients from staff-mediated transmission and 2) protecting health-workers allowing prompt treatment. In the setting of transplantation, the first is of paramount importance, being the immunosuppressed population more vulnerable to infections. As of February 11, 2020, out of 44,672 confirmed COVID-19 cases in Mainland China, 1716 (3.8%) cases were health-workers [4]. To date, 21,338 health-workers have tested positive for SARS-CoV-2 in Italy and 154 doctors (including retired ones) and 40 nurses lost their lives after being infected [5]. Shortage of personal protection devices and work overload have contributed to increase the rate of contagion within health-workers. Hosts of SARS-CoV-2 may transmit the virus while they are asymptomatic or during the incubation period, a mechanism that creates a vicious circle of in-hospital disease spread to patients and staff. Testing all the transplant staff (or, at least, those who come into contact with transplanted patients) could mitigate the risk of in-hospital transmission at the price of increased costs and workload for already under-pressure health systems. In Italy, during the fast-growing spread of COVID-19 in March, the lack of tests did not allow to adopt such an extensive screening policy.

In addition, transplant teams are at higher risk of contagion as they might travel to high incidence areas when retrieving organs for transplantation. Some countries do not have a centralized organ retrieval system and transplant teams travel outside their regions to procure organs they will implant. A “travelling organs” policy such as in the National Organ Retrieval System in the United Kingdom or Eurotransplant in central Europe help avoid transplant teams travelling from low to high incidence regions and contain the spread within medical staff. In our region, liver transplant centers based in Rome share an organ procurement scheme to retrieve and ship organs to other centers in Italy. Most regions in Italy have implemented a regional organ sharing system which, during the COVID-19 pandemic, has been increasingly utilized.

### Timing and logistics of transplantation

Due to the relevant number of false negative viral tests, there is a consistent risk of transplanting recipients who are either asymptomatic or in the incubation phase. This mandates caution and candidates for transplantation are delayed if their condition allows to. However, countries where social distancing measures have been in place for as long as the median virus incubation time, have the opportunity to rule out possible false negative tests from recipients who have complied with the social restriction policy [6, 7].

Success of transplantation relies on optimization of time constraints. The additional time required for COVID-19 testing of donors and recipients may delay organ procurement and lower the utilization rate especially of hemodynamically unstable donors that normally require fast-track management to minimize organs damage. Machine perfusion for organ preservation is expanding in almost all solid organs transplantation, allowing extend preservation time in liver, kidney, lung and heart transplantation [8, 9]. Machine perfusion could come into help when organs need to be retrieved quickly and preserved while virus tests are processed, in particular in unstable donors with low-risk history for COVID-19.

In an effort to minimize the possibility of delays which cause prolongation of cold ischemia time, back-up transplant candidates have been called in as a routine policy by several transplant centers when issues with the first-choice candidate are anticipated. Implementing such policy during the COVID-19 outbreak could offer the possibility to quickly replace the first candidate if they turn out to be SARS-CoV-2 positive.

Remote outpatient clinics via telephone or video calls (tele-clinics) are increasingly utilized to reduce hospital congestion and seminal experiences in kidney transplantation have registered even higher attendance rates than conventional clinics in selected patients [10]. Converting a proportion of outpatient clinics appointments to tele-clinics may reduce transplant population exposure to the virus. Numbers of visits (even tele-visits) can be reduced selecting only those patients with new symptoms or active issues, delaying well-being ones. A policy of remote management of immunosuppression by testing immunosuppressant level in local laboratories (then transmitted electronically) can be encouraged, thus relieving the workload on transplant centers.

### Virus tests and transplantation

In transplant services, a delay or failure to diagnose SARS-CoV-2 infection in a donor may potentially produce disastrous consequences for the recipient and also increase the risk for health-workers [11]. In this context, the role of in vitro diagnostics is crucial to screen donors and recipients. An appropriate diagnostic strategy for the detection of virus infection involves collecting the correct specimen from the patient at the right time and performing an accurate and rapid laboratory test (Table 2).

**Table 2:** Diagnostic tests available in Italy to detect SARS-CoV-2 infection

Method	Type of specimen required	Time required for assay	Advantages	Limits
**Real time reverse transcription-polymerase chain reaction**	Respiratory and non-respiratory tract specimens	5–8 h	Gold standard for the etiological diagnosis; high sensitivity and specificity; high safety	Complex protocol; overcoming of the throughput capacities of the laboratories with diagnostic delays; not suitable for decentralized point-of-care
**Direct amplification real-time reverse transcription-polymerase chain reaction. Diasorin Simplexa™**	Nasopharyngeal swabs	1 h	High sensitivity and specificity; simple protocol with all in one reagent; rapid response; high safety; suitable for decentralized point-of-care	For emergency use authorization only; Limited literature data; Limited to laboratories certified to perform high complexity tests
**Solid phase immuno-chromatographic assay for the detection of IgG and IgM antibodies to SARS-CoV-2.**	Whole blood, serum or plasma	5–15 min	No equipment needed; rapid response; suitable for decentralized point-of-care; good sensitivity and specificity; suitable for identifying asymptomatic patients and for screening	Not recommended as first line test for the diagnosis of acute viral infection; prone to ‘cross reactivity’; few reports about serological assay in detection of SARS-CoV-2; uncertain timing of antibodies development

#### Reverse transcription-polymerase chain reaction

The gold standard technique for detecting the SARS-CoV-2 infection is the real-time polymerase chain reaction (RT-PCR). This test has the advantage that the primers required can be produced as soon as the viral sequence is known. RT-PCR provides high levels of diagnostic sensitivity and specificity but the test protocol of nucleic acid amplification is complex and requires specialized instruments and technicians [12]. Although SARS-COV-2 RNA has been detected from a variety of respiratory sources, US Centers for Disease Control and Prevention recommends collecting only the upper respiratory NPS [13]. This indication is in accordance with Wang et al., that reported good detection rates of SARS-CoV-2 RNA in NPS (63% of the examined samples) [14]. SARS-CoV-2 RNA has been also detected from feces and blood specimens, although less reliably than from respiratory specimens. Higher viral loads have been detected soon after symptoms onset; thus, respiratory specimens should be collected within the first 7 days. Missing the time-window of viral replication can cause false negative results [15, 16]. Several RT-PCR protocols for the detection of SARS-CoV-2 RNA have been released by the World Health Organization and nowadays are widely standardized. However, work overload and logistic difficulties to ship samples to the few specialized centers, lead to significant delays in response time (up to 4–5 days in remote hospitals) [17]. This has caused issues in transplant services where rapid tests are needed to accelerate clinical decision-making. Several new generation real-time RT-PCR protocols for the detection of SARS-COV-2 RNA have been recently developed. These assays are suitable for decentralized point-of-care use and allow obtaining reliable results within 1 h (actual state-of-the-art detection methods). One of these, Simplexa™ COVID-19 Direct (DiaSorin Molecular LLC, CA) received the FDA’s emergency use authorization and it is nowadays available in Italy. Simplexa™ incorporate nucleic acid extraction, amplification and detection together into an integrated system ensuring a simple, safe and highly qualitative test [18–20].

#### Serology

A recent study reported acute antibody responses to SARS-CoV-2 in 285 patients and clarified that antibodies produced during the course of infection by symptomatic and asymptomatic patients can aid to the diagnosis of COVID-19 [21]. Immunoassays for detection of SARS-COV-2 immunoglobulin (Ig) M and IgG antibodies have proven to be highly specific and sensitive providing diagnostic evidence of infection in a few minutes. Moreover, the use of serology rapid tests could facilitate the diagnosis of SARS-CoV-2 infections when the molecular assays were performed unsatisfactorily [22, 23]. Several companies, driven by the growing demand of healthcare systems started to produce rapid immunoassays for SARSCoV-2. The majority of these are solid phase immunochromatographic assays for the qualitative and differential detection in human whole blood, serum or plasma of IgG and IgM antibodies to SARS-CoV-2. Although the manufacturers guarantee an accuracy close to 100%, doubts exist in the scientific community about the time kinetics of humoral response and for the potential cross reactivity with other coronaviruses [24]. In our opinion, active surveillance with rapid serological tests may prove a good option for the screening of asymptomatic donors and recipients.

### Transplant benefit during the pandemic

Limited resources allocation is the mainstay of patient care during catastrophes. When multiple casualties present at the same time, patients are triaged and treatments offered based on the chance of success. With the growing COVID-19 pandemic, the capacity of many intensive care units (ICU) has been saturated, which forced physicians to adopt a strict selection of patients who can be treated. Transplantation has always faced the issue of limited resources due to the scarcity of donors and the growing demand of organs. In liver transplantation, the concept of transplant benefit has gained wide acceptance in the last decade, in an effort to guarantee equity during organs allocation, counterbalancing the principles of utility (recipients with the highest chances of a good outcome) and urgency (recipients with the biggest need of transplantation) [25, 26].

The widespread of COVID-19 has already caused a drastic reduction in organ donation and this is predicted to aggravate further in the next months. Times of further restraints stimulate reconsidering principles of allocation and adopt a pragmatic approach based on the available resources. A drop in the availability of blood products due to the reduction in blood donors has been registered too. Restricting transplants only to the sickest recipients (unbalancing towards the “urgency” principle) could address the need of patients at imminent risk of death from end-stage organ failure. However, it is not known how this will increase mortality rates on the waiting list for all other patients who are delayed (i.e., those at “the bottom of the list”). As an example, patients with model for end-stage liver disease (MELD) of 30 have a 62% mortality rate without liver transplantation at 3 months while the rate drops to 25% with a MELD of 20. On the contrary, privileging liver transplant candidates with higher chances of success and therefore shorter hospital stay and lower consumption of blood transfusion (unbalancing towards the “utility” principle) would reduce the workload on ICUs, at the price of excluding the sickest candidates. Liver transplant recipients with MELD ≥30 have been shown to require about double the amount of perioperative blood transfusion and days of ICU stay compared to patients with MELD < 30 [27]. As happened in the past, it should be noted that waitlisted patients might be reluctant to undergo a transplant during the course of epidemics, especially those whose disease is not as severe to threaten life in the short-term [28].

A “phased approach” to decreasing transplant activity has been proposed, with varying degrees of reduction depending on resource availability [29]. In addition, for the continuation of a transplant programme, a “clean path” within the ICU has to be maintained and not all hospitals might be in a condition to offer it.

During the SARS outbreak in 2003 some transplant centers closed their activity temporarily and donor assessment guidelines were developed to mitigate the risk related to donor selection [30]. During the Ebola epidemic in 2014, the specifics of travel history of potential donors were thoroughly assessed by the organ procurement organizations. At that time, the high lethality of Ebola kept the number of affected people relatively low and the impact on organ donation was contained. The lack of effective treatments for Ebola stimulated the ethical debate around the value of the informed consent to transplantation in times of epidemics: a recipient might be willing to accept the risk of infection to gain the benefit of a new organ, however this does not contemplate the risk of infection spread to health-workers [31].

In the United Kingdom, the national authority for transplantation has released clinical advice on donation acceptance criteria (deceased donors will be considered only if < 50 and < 60 years of age respectively for circulatory- and brain-dead donors). Most non-lifesaving transplant programmes such as pancreas and living-donor kidney have been put on hold [32]. In Switzerland, almost all non-lifesaving transplants have been suspended. Other countries have advised in favor of a case-by-case decision on both donation and transplantation, depending on local conditions.

So far, most countries have reported a heterogeneous distribution of COVID-19 across their regions, with *foci* of high incidence of contagion causing major disruption to social life and healthcare. In a recently published article, Michaels et al. suggested to redistribute patients on the waiting list in endemic regions to less affected areas [33]. Such approach offers the advantage of not penalizing patients on the waiting list only because of their geographical distribution, however, in a rapidly changing scenario, less affected areas may need to keep their resources available for possible sudden increases in hospital beds demand.

## Conclusions

COVID-19 pandemic is an unprecedented life-changing crisis causing disruption in all the aspects of social life, especially for the wealthier economies of the world. As our health systems are built around patient-centered care, a cultural switch towards society over individual benefit seems mandatory in order not to run out of resources and guarantee the survival of our communities [34]. Stringent measures have been put in place to control the disease spread. Transplantation is one of the biggest advances in medical care and achievements in human history, a noble discipline that has crossed dangerous paths for the sake of its development. In this time of global crisis, the whole transplant community is called to join forces and develop strategies to mitigate risks and continue delivering the best possible results with the available resources to the multitude of patients awaiting organs from all over the world.

## Data Availability

The data used and analyzed during the current study are extrapolated and available from the cited articles as listed in the “Reference” section. If requested by the editors, we will provide the data and information on which the conclusions of this manuscript are based.

## Abbreviations

COVID-19: Coronavirus disease-19
ICU: Intensive care unit
Ig: Immunoglobulin
MELD: Model for end-stage liver disease
MERS: Middle East respiratory syndrome
NPS: Naso-pharyngeal swab
RNA: Ribonucleic acid
RT-PCR: Reverse transcription-polymerase chain reaction
SARS-CoV2: Severe acute respiratory syndrome Coronavirus 2
